# Centrosome clustering and cyclin D1 gene amplification in double minutes are common events in chromosomal unstable bladder tumors

**DOI:** 10.1186/1471-2407-10-280

**Published:** 2010-06-11

**Authors:** Javier del Rey, Esther Prat, Immaculada Ponsa, Josep Lloreta, Antoni Gelabert, Ferran Algaba, Jordi Camps, Rosa Miró

**Affiliations:** 1 Departament de Biologia Cellular Fisiologia i Immunologia, Institut de Biotecnologia i de Biomedicina, Universitat Autònoma de Barcelona, 08193, Bellaterra, Spain; 2 Departament de Patologia, Hospital del Mar, IMAS, Universitat Pompeu Fabra, Passeig Marítim 25-29, Barcelona 08003, Spain; 3 Departament d'Urologia, Hospital del Mar, IMAS UAB, Passeig Marítim 25-29 08003, Barcelona, Spain; 4 Departament de Patologia, Fundació Puigvert, Universitat Autònoma de Barcelona, Cartagena 340-350, 08025 Barcelona, Spain; 5 Genetics Branch, Center for Cancer Research, National Cancer Institute/NIH, 50 South Drive, Bethesda, MD 20892, USA

## Abstract

**Background:**

Aneuploidy, centrosome abnormalities and gene amplification are hallmarks of chromosome instability (CIN) in cancer. Yet there are no studies of the *in vivo *behavior of these phenomena within the same bladder tumor.

**Methods:**

Twenty-one paraffin-embedded bladder tumors were analyzed by conventional comparative genome hybridization and fluorescence *in situ *hybridization (FISH) with a cyclin D1 gene (*CCND1*)/centromere 11 dual-color probe. Immunofluorescent staining of α, β and γ tubulin was also performed.

**Results:**

Based on the CIN index, defined as the percentage of cells not displaying the modal number for chromosome 11, tumors were classified as CIN-negative and CIN-positive. Fourteen out of 21 tumors were considered CIN-positive. All T1G3 tumors were included in the CIN-positive group whereas the majority of Ta samples were classified as CIN-negative tumors. Centrosome clustering was observed in six out of 12 CIN-positive tumors analyzed. *CCND1 *amplification in homogeneously staining regions was present in six out of 14 CIN-positive tumors; three of them also showed amplification of this gene in double minutes.

**Conclusions:**

Complex *in vivo *behavior of *CCND1 *amplicon in bladder tumor cells has been demonstrated by accurate FISH analysis on paraffin-embedded tumors. Positive correlation between high heterogeneity, centrosome abnormalities and *CCND1 *amplification was found in T1G3 bladder carcinomas. This is the first study to provide insights into the coexistence of *CCND1 *amplification in homogeneously staining regions and double minutes in primary bladder tumors. It is noteworthy that those patients whose tumors showed double minutes had a significantly shorter overall survival rate (p < 0.001).

## Background

Malignant tumors typically arise from multiple events within the developing cancer cells. Genetic damage is a hallmark of malignant cells and plays a key role in both the initiation and the progression of tumorigenesis [1].

Bladder cancer, along with most solid tumors, is characterized by multiple numerical and structural chromosome aberrations which in general associate with progression [2,3]. Amplification of 11q13 involving cyclin D1 gene (*CCND1*) is among the most common sites of gene amplification in T1-T2 high grade tumors [4-6]. Cyclin D1 plays an important role in cell cycle, binds to cyclin dependent kinases (CDK4/6), and promotes phosphorylation of RB1, orchestrating progression through the G1 restriction point.

Gene amplification involving oncogenes, a common mechanism to overexpress cancer-related genes, might be present in cancer cells as double-minute chromosomes (DMs) or homogeneously staining regions (HSRs). DMs are circular extrachromosomal autonomously-replicating DNA fragments lacking a centromere. HSRs are amplified intrachromosomal sequences that may be located in the same region of the amplified gene or in another chromosomal region [7]. The 11q13 amplicon is generally located at the same chromosome region of the single-copy genes involved (*CCND1*, etc.) [8]; other amplifications, such as those involving *MYCN *in neuroblastomas, are inserted in several places in the genome other than chromosome 2, where *MYCN *gene is mapped [9,10].

Numerical chromosome instability (CIN), which occurs very frequently in cancer cells [11], contributes to aneuploidy and plays a critical role in tumorigenesis as a key element of genomic instability [11,12]. Chromosome missegregation resulting from the deregulation of the spindle checkpoint is thought to be a potential cause of CIN. However, the molecular basis of this causative relation remains largely unknown [13]. The centrosome, a major microtubule-organizing center in animal cells, plays a vital role during mitosis as a spindle pole, and is crucial for accurate chromosome segregation to daughter cells [14]. In previous studies, centrosome amplification, defined as an increase in the centrosome number, has been identified in many different tumors, including bladder cancer [15,16]. In addition, centrosome amplification has been recently shown to initiate tumorigenesis in flies [17]. Several studies have demonstrated that centrosomal abnormalities and chromosome copy-number heterogeneity frequently co-exist in bladder tumor cells [18-20]. More recently, Jin et al. [21] found that multipolar mitosis and anaphase bridges are common, often concurrent, mitotic abnormalities in urothelial carcinomas, both *in vivo *and *in vitro*. The same authors identified several types of chromosome segregation abnormalities, including telomere dysfunction, sister-chromatid non-disjunction, and supernumerary centrosomes in urothelial cancer cell lines. These studies strongly support the hypothesis that CIN is present in bladder carcinomas.

The aim of this study was to describe how *CCND1 *amplicons and chromosome 11 copy number heterogeneity represent *in vivo *features of chromosomal instability in superficial bladder carcinomas. To that end, 21 paraffin-embedded cancer tissue samples were analyzed using comparative genomic hybridization (CGH) and fluorescence *in situ *hybridization (FISH). In seeking a basis for the chromosomal heterogeneity, we investigated centrosome and mitotic spindle integrity by immunofluorescent staining. Our results demonstrate, for the first time, that *CCND1 *amplification in DM and HSR could co-exist in the same bladder tumor. A correlation between HSR fragmentation and the appearance of DMs, which were subsequently eliminated by micronuclei extrusion, was also observed. Interestingly, we found that those patients whose tumors showed *CCND1 *amplification in DMs had a significantly shorter overall survival rate. Finally, the correlation between chromosome instability and centrosome abnormalities showed that the coalescence of centrosomes into two functional spindle poles was common in unstable bladder tumors.

## Methods

### Samples

Twenty-one formalin-fixed and paraffin embedded bladder-tumor samples were obtained from the Fundació Puigvert and Hospital del Mar of Barcelona. Tumor stage and grade were defined according to WHO criteria [22]. All tumors were superficial or minimally invasive (nine pTa, 12 pT1). Of 21 cases, six were grade 1, eight were grade 2 and seven were grade 3. Clinical and histopathological data are indicated in table 1. In one patient (case U-443), the first recurrence of the tumor and the penile and inguinal lymph node metastases were also studied.

**Table 1:** Patient characteristics and study results

	Case	Age/Sex	Stage/Grade	S/M	CIS	Modal number Chr 11	CIN index	CGH 11q13	FISH *CCND1*	Subpop	SC (%)	C size (μm)	AC	MS (%)	Recurrence (months)	Survival	CRD
**CIN negative**	**U-400**	45/♂	TaG1	S	-	2	17.64	normal	normal	No	0	0.8	-	0	0	>6 years	No
	**U-114**	63/♀	TaG1	S	-	2	20	normal	normal	No	3.8	1.10	-	0	1;(19)	>6 years	No
	**U-408**	66/♂	TaG2	S	+	2	20.18	normal	normal	No	0	0.98	-	0	3;(4),(60),(8)	>6 years	No
	**U-814**	68/♂	T1G2	M	-	2	23.97	gain	gain	No	1	0.63	-	0	5;(8),(15),(34),(43),(3)	>5 years	No
	**U-373**	52/♀	TaG2	M	-	2	25.9	normal	normal	No	0	0.9	-	0	1;(37)	>6 years	No
	**U-906**	58/♂	TaG1	S	-	2	25.41	normal	normal	No	1	0.81	-	0	1;(67)	>5 years	No
	**U-433**	68/♂	TaG1	S	+	2	28.75	normal	normal	No	?	?	?	?	1;(28)	>6 years	No

**Moderate CIN**	**U-611**	54/♂	T1G3	S	+	2	33.7	normal	normal	No	0	2.04	+	0	0	>42 months	No
	**U-443**	62/♂	T1G3	S	-	2	34.16	amp	HSR	Yes (3)	0	0.98	-	0	3;(17),(13)*,(34)*	67 months	Yes
	**U-955**	48/♂	TaG1	S	-	2	37.91	normal	normal	No	0	0.8	-	0	0	>6 years	No
	**U-089**	45/♂	TaG2	S	-	2	43.33	normal	normal	No	5	1.21	+	0	2;(8),(13)	>5 years	No
	**U-150**	60/♂	T1G2	S	-	2	43.51	amp	HSR, DM	Yes (2)	21	4.88	+	0	5;(10),(17),(9),(6),(7)	54 months	Yes
	**U-617**	67/♂	TaG1	S	-	2	47.5	normal	normal	No	?	?	?	?	2;(9),(11)	>6 years	No
	**U-013**	73/♂	T1G2	S	+	2	54.83	normal	normal	No	4	1.06	-	0	0	>41 months	No
	**U-532**	51/♂	T1G3	M	+	2	57.14	normal	gain	No	38	0.68	+	7	0	>6 years	No

**High CIN**	**U-076**	67/♂	T1G3	M	+	2	60.41	amp	HSR, DM	Yes (2)	20	2.22	+	17	3;(9),(4),(10)	31 months	Yes
	**U-866**	40/♂	T1G3	M	+	3	65.68	normal	gain, HSR, DM	Yes (3)	0	5.69	+	0	-	36 months	Yes
	**U-364**	73/♂	T1G3	S	-	2	67.61	normal	gain, HSR^1^, HSR^2^	Yes (3)	27	2.06	+	17	0	>3 months	No
	**U-183**	62/♂	T1G3	M	+	3	70	normal	HSR	No	5	1.18	+	0	0	>6 years	No
	**U-466**	71/♂	T1G2	S	-	4	70.66	gain	gain	No	?	?	?	?	1;(14)	>6 years	No
	**U-564**	55/♂	T1G2	S	-	2	70.68	loss	gain	No	0	2.01	+	0	2;(10),(3)	>6 years	No

**S**: single, **M**: multiple, **CIS**: carcinoma in situ, **CIN index**: percentage of cells not displaying the modal copy number for chromosome 11, **subpop**: presence and number of intratumor subpopulations, defining subpopulation as a group of cells with a distinctive chromosomal alteration (numerical or structural) at a specific area on the tumor, **amp**: amplification, **HSR**: homogeneously staining region, **HSR1,2**: different types of homogeneously staining region, **DM**: double minute, **SC**: percentage of cells displaying supernumerary centrosomes (>2 centrosomes), **C size**: average size of centrosome, **AC**: presence of abnormal centrosomes (size >2 μm or number >2 in at least 5% of cells), **MS**: percentage of cells displaying multipolar spindle, *: metastasis, **CRD**: cancer related death, **?**: data not available

### Conventional comparative genomic hybridization

For each tumor sample, DNA was extracted from four to five 10 μm paraffin sections using a Qiagen Kit: QIAamp^® ^DNA Mini Kit. Before extraction, evaluation by the pathologist determined that the proportion of tumor cells was higher than 80%. The first and the last sections were stained with hematoxylin/eosin to ensure the presence of tumor in the sections series. Comparative genomic hybridization analysis was performed according to the method described by Prat et al.[3].

### *CCND1 *amplification and CIN analysis

CIN generates intercellular numerical variation for the same chromosome within a given tumor. Fluorescence *in situ *hybridization analysis can be considered as a practical method to detect CIN in surgical specimens [23]. In this study, CEP 11 was used as the copy-number reference of chromosome 11. FISH was carried out using Spectrum Orange-labeled *CCND1 *and Spectrum Green-labeled CEP 11 (Vysis Inc., Downers Grove, IL). Five μm sections from representative tissue blocks were used in this study. Briefly, slides were placed in the oven for 30 min at 65°C and paraffin was dissolved in Xylene. Slides were boiled in EDTA 1 mM, followed by a pepsin treatment. Post-fixation was performed in 1% formaldehyde. Co-denaturation of the slide material and the probes was carried out according to the manufacturer's instructions using a Hybrite (Vysis Inc.). Hybridization took place overnight at 37°C in a moist chamber. Nuclei were counterstained with 4',6-Diamidino-2-Phenylindole (DAPI, Sigma) diluted on Vectashield antifade solution at a final concentration of 125 ng/μl. Analysis was done under a fluorescence microscope (Olympus BX-50). A minimum of 280 cells were scored in each case.

CIN index was defined as the percentage of cells not displaying the modal copy number for the studied chromosome [16,23], in this case chromosome 11. The samples were graded according to their CIN index, as negative CIN (<30% of the cells with non-modal signal number), moderate (>30% <60% of the cells) and high CIN (> 60% of the cells). FISH was also applied in order to analyze the amplification of *CCND1 *gene. Amplification was only considered when it appeared in HSR or DM forms.

### Immunofluorescent analysis of centrosome and mitotic spindle

Briefly, 5 μm sections from representative tissue blocks were de-paraffinized in xylene, and then rehydrated in ddH_2_O through graded alcohols. Slides were boiled in 1 mM EDTA buffer (pH.8) and then were incubated overnight at 4°C in PBTG solution (PBS, 0.2% BSA, 0.2% gelatin, and 0.05% Tween 20) with primary rabbit-polyclonal γ-tubulin (T3559, Sigma-Aldrich, 1:100) and mouse-monoclonal α-tubulin (T5168, Sigma-Aldrich, 1:200) and β-tubulin (T4026, Sigma-Aldrich, 1:200). Signal detection was performed applying fluorochrome-conjugated secondary antibodies (all from Jackson ImmunoResearch Laboratories): goat anti-rabbit Cy3 (diluted 1:1,000 in PBTG) and goat anti-mouse Cy5 antibody (diluted 1:1,000 in PBTG). Secondary antibodies were incubated for 1 h at 37°C. Four PBTG washes were carried out. Fixation was performed in 1% formaldehyde. Tissue sections were counterstained with DAPI and then examined under a fluorescence microscope (Olympus BX-50).

Measurements of centrosome lengths were made using the MicroMeasure v3.3 software http://www.biology.colostate.edu/micromeasure. The presence of supernumerary centrosomes was considered whenever the centrosome number was ≥3 in at least 5% of the cells. Abnormally large centrosomes (diameter ≥2 μm) were indicative of centrosome clustering.

### Statistical analysis

The statistical analysis of the data was carried out by using the SPSS software package (SPSS Inc; Chicago, IL, USA; Version 15.0). Overall survival was estimated with the Kaplan-Meier method. The survival curves were statistically compared by a log-rang test. Fisher's exact test was used to find associations. p < 0.05 was considered statistically significant.

## Results

### Classification of tumors according to the CIN index

**Figure 1:**
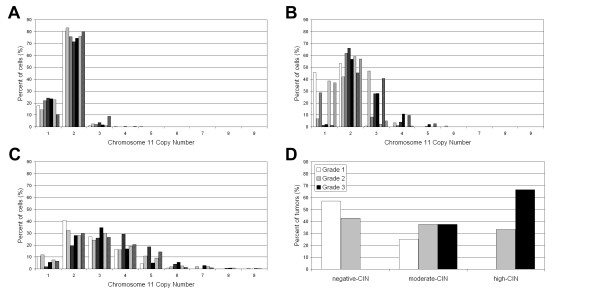
**Chromosome 11 copy number variability**. (A) Negative-CIN tumors. (B) Moderate-CIN tumors. (C) High-CIN tumors. (D) CIN index vs. tumour grade correlation.

Depending on the percentage of cells with a number of chromosome 11 different from the modal number, tumors were classified into three groups: high, moderate and negative CIN (Table 1). Samples showing an intercellular variation in the number of centromeric signals for chromosome 11 greater than 30% were deemed to have chromosomal instability. Fourteen out of 21 samples (66%) were classified into the CIN-positive group. CIN-negative tumors showed a modal number of 2 using a centromere-specific FISH probe for chromosome 11. Only a small fraction of the tumor cells showed monosomy, while trisomy was even less frequent (Figure 1A). Within the CIN-positive group, tumors with moderate CIN levels had a modal number of 2. CIN-moderate samples showed a greater chromosome 11 copy number range than CIN-negative group, with trisomy the most frequently observed (Figure 1B). The high CIN tumors had wide-ranging chromosome 11 copy numbers (Figure 1C). Monosomy was rare; the modal number was disomic in three samples, trisomic in two samples, and tetrasomic in only one sample.

There was a positive correlation between the CIN index and the tumor grade (Figure 1D). Moreover, the majority of Ta G1/G2 tumors (6/9) were included in the CIN-negative group whereas T1G3 tumors were exclusively found in high CIN (4/7) or moderate CIN (3/7) groups (Table 1).

### Centrosome defects and multipolar mitoses

In seeking a basis for the observed chromosomal heterogeneity, we investigated centrosome and spindle integrity using immunofluorescent staining. Results were obtained for 18 of the 21 samples. In general, there was a positive correlation between the CIN index and centrosome abnormalities (p < 0.005). We have used the terms supernumerary centrosomes and centrosome clustering to describe the abnormalities of the centrosomes observed in our study. Despite the fact that both terms imply the presence of extra centrosomes, we considered centrosome clustering when centrosomes could be microscopically observed as abnormally-shaped or large centrosomes and supernumerary centrosomes when they were observed individually.

**Figure 2:**
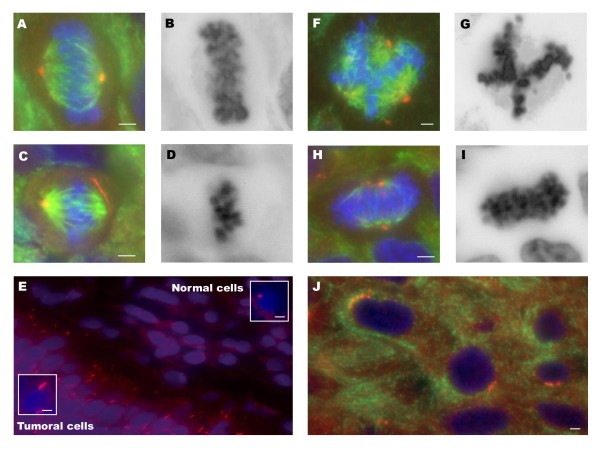
**Centrosome abnormalities**. Immunolabeling was performed for γ-tubulin (red) and α and β-tubulin (green). DNA staining was performed with DAPI (blue). Black and white images correspond to DAPI reverse staining. (A-B) Normal/bipolar spindle. (C-D) Bipolar metaphase with string-like centrosome. (E) Tumour cells with abnormally long centrosomes, close to the adjacent normal urothelium. (F-G) Multipolar spindle. (H-I) Pseudo-bipolar metaphase. (J) Tumour cells with supernumerary centrosomes. Scale bar, 3 μm.

Spindle errors were not present in samples with normal centrosome numbers (Figure 2A-B). In our study, supernumerary centrosomes were the most frequent aberration identified. The presence of enlarged centrosomes or shape aberrations such as string-like centrosomes (Figure 2C-E), which are indicative of centrosome clustering, was also frequently observed (Table 1). All the high CIN samples (n = 5) showed abnormal centrosomes. Overall, supernumerary centrosomes and centrosome clustering were found in 60% and 80% of the tumors, respectively (Figure 2F-J). In two samples (U-076 and U-364), both centrosome alterations were concomitant. In the moderate CIN group, four out of the seven samples (57%) showed abnormal centrosomes. Supernumerary centrosomes were found in three samples, while centrosome clustering was present in two. In one of them (U-150), both alterations were found simultaneously (Table 1). Centrosome abnormalities were absent in normal tissue adjacent to the tumor cells (Figure 2E). String-like centrosomes were found in three samples (U-150, U-866 and U-564) (Figure 2C-E). The longest centrosome was found in sample U-150 (7.33 μm). These extraordinarily long centrosomes were involved in the formation of bipolar spindles (Figure 2C-D, Table 1).

Multipolar and/or pseudo-bipolar mitoses were observed in three out of six samples (50%) with supernumerary centrosomes (Figure 2F-I). Coalescence of supernumerary centrosomes into two functional spindle poles was observed in all samples with enlarged centrosomes.

### *CCND1 *gene amplification: FISH *vs. *CGH

The copy number status of *CCND1 *was analyzed using FISH and conventional CGH (Table 1). *CCND1 *amplification was identified by FISH in most of the high-CIN samples (four out of six), in 25% of the moderate-CIN samples (two out of eight) and in none of the CIN negative samples. Concordance between FISH and CGH results was observed in 16 out of 21 cases (76%); all samples with disagreement between FISH and CGH results were CIN-positive. Sample U-532 showed no 11q13 gain using CGH; however, 42% of cells within this sample showed five or more copies of *CCND1 *using FISH, even though the modal number was 2. The most divergent results were found in high-CIN tumors. Three samples (U-866, U-364 and U-183) showed *CCND1 *amplification using FISH, although it was not detected using CGH. Sample U-564 showed a whole chromosome 11 loss by CGH, however more than five copies in 23% of the cells were detected using FISH. In summary, amplification of the *CCND1 *in DMs and/or HSRs was detected using FISH in six cases; amplification was detected using CGH only in three cases (U-443, U-150 and U-076).

### Intratumor cell sub-populations and *CCND1 *amplification behavior

By accurately analyzing the samples showing *CCND1 *amplification, various cell sub-populations were detected within the tumors. A cell sub-population is defined as a group of cells with a distinctive chromosomal alteration (numerical or structural) at a specific area of the tumor. Analysis of these sub-populations provided insights into the *in vivo *behavior of *CCND1 *amplification.

**Figure 3:**
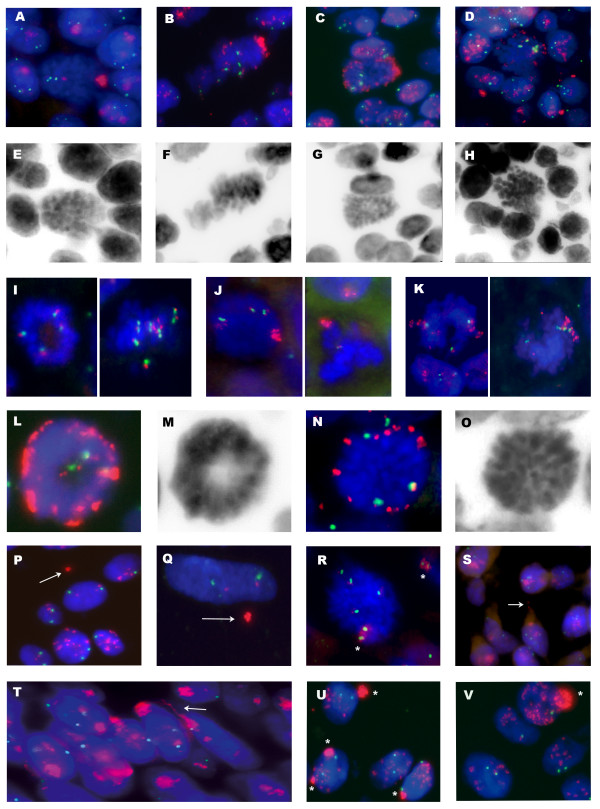
***CCND1 *amplification behaviour in bladder tumors**. FISH identification of chromosome 11 centromere (green) and *CCND1 *gene (red) in paraffin-embedded tumors. DNA staining was performed with DAPI (blue). Black and white images correspond to DAPI reverse staining. (A-H) Metaphasic cells showing the proposed sequence of 11q13 amplicon fragmentation from HSRs to DMs. (I-K) Sample U-364 showed a complex pattern of *CCND1 *amplification. Three sub-populations were detected in this sample. (I) Sub-population with gain of whole chromosome 11. (J) Sub-population containing HSR with high-level amplification of *CCND1 *(K) Sub-population containing amplification of *CCND1 *and undetermined flanking material in HSR. (L-O) Peripheral location of DMs in metaphasic cells. (P-Q) *CCND1*-positive micronuclei, see arrows. In Q, note the elimination in the micronucleus of whole *CCND1 *copies, except those attached to the centromere. (R) Metaphasic cells containing a dicentric chromosome with two centromeric signals of chromosome 11 and *CCND1 *amplification, see asterisks. (S and T) *CCND1 *with HSRs appears to be forming internuclear bridges, see arrows. (U and V) Nuclear blebs as nuclear protrusions with high *CCND1 *signal.

Discrete cell sub-populations were found in three samples (U-076, U-866 and U-364) with a high CIN index and in two (U-443, U-150) with a moderate CIN index (Table 1). Sample U-076 had two sub-populations: one showed *CCND1 *amplification in HSRs, while the other showed amplification in DMs, probably due to excision from HSRs. There were also intermediate conformations, as shown in Figure 3A-H. DMs and HSRs are readily identifiable at the metaphase stage, but can also be distinguished in interphase nuclei. While HSRs are seen as a compact and distinct signal, DMs showed a diffuse signal. The co-existence of DMs and HSRs was also detected in two other samples (U-150 and U-866).

Sample U-364 showed a complex pattern of *CCND1 *amplification. Three sub-populations were detected in this sample, including one with whole chromosome gain up to nine copies (Figure 3I); two sub-populations showed *CCND1 *amplification as two HSRs, both of which varied significantly in terms of structure and size. One sub-population generated an amplicon with a high *CCND1 *copy number, which was viewed during the metaphase stage as a long *CCND1*-positive HSR (Figure 3J). The other sub-population showed a different amplification pattern, with fewer *CCND1 *copies, each of which was surrounded by some undetermined genomic material, as seen during the metaphase stage (Figure 3K).

In patient U-443, it was possible to analyze the behavior of the amplicon over a period of time. *CCND1 *amplification as a compact HSR was detected in the primary tumor, its recurrence and penile metastasis (after 17 and 30 months, respectively). However, diffuse HSR amplification and a small fraction of cells with DMs were observed in an inguinal lymph node metastasis detected 34 months after the penile metastasis. These results suggest that HSRs could remain stable during long periods before giving rise to DMs during a late-metastasis. (The patient died three months following the metastasis).

It is noteworthy that those patients, whose primary tumors showed DMs had cancer-related deaths. The Kaplan-Meier analysis showed that these patients (U-150, U-76 and U-866) had a significantly shorter overall survival rate than patients without DMs in their primary tumors (including U-443) (p < 0.001). Therefore we concluded that high heterogeneity samples showed different populations with amplification of *CCND1 *and it correlates with clinical features.

### DMs distribution on the metaphase plate, micronuclei, internuclear bridges and nuclear blebs

This analysis was carried out on samples showing *CCND1 *gene amplification in DMs (samples U-150, U-076 and U-866) (Table 2). DMs containing *CCND1 *were predominantly located in the peripheral region of the metaphase plate (Figure 3L-O) (Table 2). Metaphases were analyzed in paraffin-embedded tissue sections, allowing the identification of DMs containing *CCND1 *non-randomly located at the periphery during metaphase in bladder primary tumors.

**Table 2:** DM localization on metaphase plate and micronuclei frequencies

	**Peripheral localization of ** **DMs**		***CCND1 *Positive ** **MN**		**CEP11 positive ** **MN**		**Total MN**	
				
**Sample**	**per ** **metaphase**	**per 100 ** **metaphase**	**per ** **nuclei**	**per 100 ** **nuclei**	**per ** **nuclei**	**per 100 ** **nuclei**	**per ** **nuclei**	**per 100 ** **nuclei**
				
**U-150**	22/25	88.00%	28/472	5.93%	1/472	0.21%	62/472	13.14%
**U-076**	27/36	75.00%	19/477	3.98%	1/477	0.21%	48/477	10.06%
**U-866**	15/20	75.00%	10/412	2.43%	10/412	2.43%	42/412	10.19%

**DM**: Double minute, **MN**: Miclonuclei, **CEP**: Centromere

All samples with DMs showed nuclear blebs and micronuclei, whereas the samples without DMs did not. The size of the micronuclei varied from 5-30% of nuclear volume. The number of micronuclei per 100 cells varied from 10-13 (Table 2). In samples U-150 and U-076, a significant percentage of micronuclei were *CCND1*-positive (45% and 40% respectively) (Figure 3P-Q). Approximately 2% of the micronuclei in these samples were positive for chromosome 11 centromere. In sample U-866, there were 10 micronuclei per 100 cells, being 24% of them *CCND1*-positive. This tumor had a higher proportion of micronuclei positive for centromere 11 (24%), indicating a very high level of aneuploidy, compared with the previous two samples (U-076 and U-150). The complete removal of DMs by micronucleus extrusion, giving rise to cells with two or three *CCND1 *gene copies, was also observed in a small number of cells (Figure 3P-Q). The presence of metaphase dicentric chromosomes, internuclear bridges, and nuclear blebs positive for the *CCND1 *amplification demonstrated the ongoing chromosomal instability observed in high CIN bladder primary tumors (Figure 3R-V).

## Discussion

In the present study, the gene copy number variation analysis of *CCND1 *in formalin fixed paraffin embedded tissue sections revealed a complex and unprecedented pattern of cellular behavior in non-muscle invasive bladder tumors. Our results suggested that copy number changes of *CCND1 *could be used as a biomarker to detect chromosome instability in bladder cancer. Bladder tumors were classified according to the CIN index, and we have shown a positive correlation between high heterogeneity, centrosome abnormalities and *CCND1 *gene amplification.

A positive correlation between the level of chromosomal instability and the tumor grade was identified; this phenomenon was previously described in bladder cancer [16]. Focusing exclusively on chromosome 11, the present study classifies the majority of Ta tumors as stable (CIN-negative group). These results are in agreement with the 2004 WHO classification that distinguishes two different entities in non-muscle invasive bladder tumors: one (Ta low-grade G1/G2) genetically stable in which gene amplifications are rare, and the other (T1 high-grade) with a high degree of genetic instability including high level amplifications [24]. Despite the low number of samples analyzed, it is remarkable that our classification of tumors according to their CIN index included all T1G3 samples in the CIN-positive group. CIN-negative group was just composed of grade 1 and grade 2 tumors, being all but one Ta. *CCND1 *amplification was exclusively observed in CIN-positive samples, suggesting that *CCND1 *might be involved in the generation of centrosomal abnormalities [25]. Moreover, we showed amplification of *CCND1 *as DMs in three CIN-positive samples. To our knowledge, this is the first report in the literature to show genomic amplification of *CCND1 *as DMs in bladder tumors.

The differences between FISH and metaphase CGH results for the CIN-positive samples highlight intratumoral heterogeneity. CGH detected the dominant genomic alterations present in at least some 60% of the tumor population [26]; however, it did not detect either the alterations that appear in a small number of cells or ongoing chromosomal instability. The association of DMs, centrosome aberrations and intercellular CIN observed in this study may indicate that the CIN phenotype does not become the major clonal population in bladder cancer.

As expected, centrosome amplification is correlated with CIN. Approximately, 75% (9/12) of CIN-positive samples and none of the CIN-negative samples showed centrosomal abnormalities. During the analysis of metaphase figures, multipolar and pseudo-bipolar spindles were identified in some CIN-positive tumors with supernumerary centrosomes. The presence of extra centrosomes within tumor cells might be deleterious as multipolar mitosis may generate sufficient high levels of aneuploidy to compromise cell viability. Several cancer cell lines overcome this problem by clustering their extra centrosomes at the two poles of the spindle, thus ensuring bipolar chromosome segregation [27-29]. This phenomenon was observed in some samples in the present study showing abnormal large centrosomes and bipolar spindles, what confirms that centrosome clustering occurs in bladder cancer. It is interesting to note that bipolar spindles were also observed in three CIN-positive samples displaying a string-like centrosome similar to what was observed by Pihan et al. [30] in malignant tumors and tumor-derived cell lines.

In addition, the high chromosomal instability observed in our samples with centrosome clustering suggests that other factors might cause chromosomal instability. In fact, chromosome lagging, defined as a delayed movement of one chromatide in anaphase, was observed when anaphasic cells were studied. This is consistent with studies carried out by Thompson and Compton [31] on human cell lines, where the authors identified defective kinetochore-spindle attachments leading to anaphase lagging as a cause of chromosome missegregation. Recently, Ganem et al. [32] demonstrated that extra centrosomes alone are sufficient to promote chromosome missegregation during bipolar cell division. According to these authors, cells passing a transient multipolar spindle intermediate accumulate merotelic kinetochore-spindle attachment errors before centrosome clustering and anaphase.

DNA sequence amplification is one of the hallmarks of genomic instability in cancer. The target genes driving the 11q13 amplicon have been extensively reported, and at least four cores of amplification have been established in breast cancer [33,34]. However, the evolution of this amplicon in tumor cells remains unclear. The 11q13 amplicon is usually located in the same chromosomal region as the amplified target gene [7,8,35,36]. In our study, HSRs were usually located in the same chromosomal region as the amplified target genes, thus strongly supporting the hypothesis that the 11q13 amplicon is of intrachromosomal origin [8]. The presence of dicentric chromosomes and anaphase bridges in cell populations undergoing amplification is consistent with the role of the breakage-fusion-bridge (BFB) cycle in explaining intrachromosomal amplifications [37]. In the present study, the HSR-bearing chromosome 11 was often observed to be involved in nucleoplasmatic bridges and dicentric chromosomes.

Nevertheless, several mechanisms for the genesis of extrachromosomal amplifications (i.e., DMs) have been proposed [38]. A yeast model system was used to demonstrate that hairpin-capped double-strand breaks occurring at the location of human Alu-quasipalindromes trigger both DM and HSR gene amplification. According to this model, the nature of the amplicons depends on the chromosomal location of the amplified gene relative to double-strand break formation [39]. Within our sample set, the co-existence of *CCND1 *amplification in DMs and HSR is noteworthy. In tumor samples with both types of amplification, metaphasic cells with both HSR and DMs were observed in the transition zone between the HSR and DM carrier cell sub-populations. These data demonstrate a striking correlation between the presence of DMs and the observed fragmentation of HSR, thus suggesting a possible mechanism for excising amplified sequences in HSRs, giving rise to DMs. A similar phenomenon has been described in human cell lines with dihydrofolate-resistance gene amplification [40,41]. Our findings strongly suggest that the same mechanism operates in tumor cells *in vivo*. Moreover, as seen in patient U-443, fragmentation of the HSR might occur in a metastatic form, after remaining stable for a long period of time in the primary tumor.

All samples with DMs also exhibited micronuclei; approximately 50% of them were *CCND1*-positive. The fact that the *CCND1 *signal was not present in the remaining 50% of the micronuclei indicates that other genomic regions were being actively eliminated from these cells. These findings suggest that micronuclei extrusion could induce rapid and dramatic changes, not only in the *CCND1 *gene with DMs, but also in other acentric fragments or even affecting whole chromosome copy numbers, therefore exacerbating genomic instability. The removal of amplified *CCND1 *sequences by micronuclei extrusion in bladder tumor cells was consistent with results reported by Valent et al. [42] regarding DMs containing *MYCN *neuroblastoma. Furthermore, in the present study, some cells showed one to three copies of the *CCND1 *on chromosome 11, but with an adjacent *CCND1*-positive micronucleus, suggesting that in some cells the normal copy number for this gene is restored by DMs extrusion.

It is known that centrosome amplification is a source of CIN, as are chromosomal lagging and micronuclei formation. Centrosome clustering partially reduces chromosomal instability [28], and increases cell viability by avoiding multipolar mitosis. Our observations that centrosome clustering is a common feature of chromosomally unstable bladder tumors, and the appearance of new drugs that specifically target centrosome clustering, such as griseofulvin [43], highlights the importance of further studying the role of centrosome abnormalities in bladder cancer.

## Conclusions

The present study describes the *in vivo *behavior of *CCND1 *amplification in chromosome unstable T1 bladder tumors. We also demonstrate that the coalescence of centrosomes into two functional spindle poles is a common feature of these tumors.

Our study is the first report in the literature regarding the simultaneous *CCND1 *amplification in DM and HSR in bladder cancer cells. Our findings suggest a striking correlation between HSR fragmentation and the appearance of DMs which subsequently are removed by micronuclei extrusion. Of interest, we found that only those patients whose tumors showed *CCND1 *amplification in DMs had a significantly shorter overall survival rate. Further studies in a larger sample size should be necessary in order to confirm our results.

Coalescence of supernumerary centrosomes was observed in 80% of the most unstable tumors, highlighting the importance of this phenomenon in bladder cancer.

Data presented here contribute to the understanding of the *in vivo *chromosome behavior of bladder tumor cells, and show how its complexity could be analyzed by FISH on paraffin embedded tumors as if snapshots of what occurs in the tumor at the time of surgical removal had been taken.

## List of abbreviations

CIN: chromosome instability; *CCND1: *cyclin D1 gene; DM: double-minute; HSR: homogeneously staining region; CGH: comparative genomic hybridization; FISH: fluorescence in situ hybridization.

## Competing interests

The authors declare that they have no competing interests.

## Authors' contributions

JdR carried out all the experimental studies, participated in design of the study, analysis and interpretation of data and drafted and revised the manuscript. EP participated in CGH analysis and helped to draft the manuscript. IP participated in FISH analysis. JL, AG and FA were involved in acquisition and interpretation of data. JC was involved in drafting and revising the manuscript. RM conceived of the study and participated in its design and coordination, was involved in drafting and revising the manuscript. All authors read and approved the final manuscript.

## Pre-publication history

The pre-publication history for this paper can be accessed here:

http://www.biomedcentral.com/1471-2407/10/280/prepub
